# Differential effects of locally and systemically administered soluble glycoprotein 130 on pain and inflammation in experimental arthritis

**DOI:** 10.1186/ar3079

**Published:** 2010-07-13

**Authors:** Michael K Boettger, Johannes Leuchtweis, Diana Kümmel, Mieczyslaw Gajda, Rolf Bräuer, Hans-Georg Schaible

**Affiliations:** 1 Institute of Physiology I/Neurophysiology Jena University Hospital - Friedrich Schiller University, Teichgraben 8, D-07743 Jena, Germany; 2 Institute of Pathology, Jena University Hospital - Friedrich Schiller University, Ziegelmühlenweg 1, D-07743 Jena, Germany

## Abstract

**Introduction:**

Interleukin-6 (IL-6) is a key player in systemic arthritis, involved in inflammation and joint destruction. IL-6 signalling has also been revealed in nerve cells. Recently, IL-6 and in particular IL-6 together with its soluble IL-6 receptor (sIL-6R) were shown to induce a long-lasting robust sensitization of joint nociceptors for mechanical stimuli which was difficult to reverse, suggesting that IL-6 signalling plays a significant role in the generation and maintenance of arthritic pain. Here we tested in a preclinical model of arthritis, antigen-induced arthritis (AIA) in the rat, whether systemic or local neutralization of IL-6/sIL-6R complexes with soluble glycoprotein 130 (sgp130) alters arthritic pain and how sgp130 influences the inflammatory process in AIA.

**Methods:**

Rats with AIA were either treated with sgp130 or saline intra-peritoneally or intra-articularly (each group n = 9). Then, pain-related and locomotor behaviour, as well as joint swelling, were measured during an observation period of 21 days, followed by histopathological end-point analysis for inflammatory and destructive changes.

**Results:**

A single intra-articular application of sgp130 at the time of AIA induction barely reduced the development of AIA, but significantly attenuated pain-related behaviour, that is, primary mechanical hyperalgesia in the acute phase of AIA. By contrast, repeated systemic application of sgp130 after onset of AIA only slightly attenuated pain at a late stage of AIA. None of the treatments reduced secondary hyperalgesia. Furthermore, in the present study joint destruction at 21 days was significantly attenuated after intra-articular sgp130 treatment, but not after systemic sgp130.

**Conclusions:**

In addition to its role in chronic inflammation, IL-6 in the joint plays a significant role in the generation and maintenance of arthritic joint pain at acute and chronic stages of AIA. The particular effectiveness of intra-articular injection of sgp130 indicates, first, that IL-6/sIL-6R in the inflamed joint, rather than circulating IL-6/sIL-6R, is responsible for the generation of hyperalgesia, and, second, that early neutralization of IL-6/sIL-6R is particularly successful in producing antinociception. Furthermore, neutralization of IL-6/sIL-6R (and possibly other cytokines which use the transmembrane signal-transducing subunit gp130) directly at the site of joint inflammation seems to be effective in the prevention of joint destruction.

## Introduction

The cytokine interleukin-6 (IL-6) is thought to be a key player in systemic inflammation and arthritis [1], as shown, for example, by significantly attenuated antigen-induced arthritis in IL-6-deficient mice [2]. In a murine model of human tumour necrosis factor- (TNF) mediated inflammation, IL-6 was found to be particularly involved in inflammation-evoked osteoclast formation and bone erosion [3]. IL-6 signalling not only depends on the presence of IL-6 but also on various cofactors. IL-6 can bind to a membrane-bound IL-6 receptor (IL-6R) which acts in cooperation with the transmembrane signal-transducing subunit soluble glycoprotein 130 (gp130) [4,5]. Alternatively, IL-6 can bind to a soluble IL-6 receptor (sIL-6R), and the IL-6/sIL-6R complex can bind to the transmembrane signal-transducing subunit gp130 of cells which do not express the membrane-bound IL-6R, thus leading to IL-6 transsignalling [5]. In fact, in the serum, synovial fluid and synovial tissue of rheumatoid arthritis patients the concentrations of both IL-6 [6,7] and sIL-6R [8,9] are elevated. While sIL-6R acts as an agonist, circulating soluble gp130 (sgp130) acts as an antagonist, because it binds IL-6/sIL-6R complexes and thus prevents transsignalling [4,5]. In murine arthritis models, neutralization of IL-6 transsignalling by administration of sgp130 was shown to reduce inflammation [2,10-12].

In addition, it has been suggested that IL-6 plays an important role in the generation of inflammatory pain. Using electrophysiological recordings from nociceptors of the rat knee joint, we recently found that the injection of IL-6 or of IL-6 together with sIL-6R into a normal knee caused a long-lasting sensitization of nociceptive C-fibres for mechanical stimuli applied to the joint [13]. This sensitization is likely to be caused at least in part by a direct effect on the nerve fibres, because most peripheral nerve fibres were shown to express the transmembrane signal-transducing subunit gp130 [14,15]. The sensitizing effect of IL-6 was prevented by co-administration of sgp130 which binds and inactivates IL-6/IL-6R complexes [5]. Interestingly, however, sgp130 did not reduce the enhanced mechanosensitivity when it was administered into the joint one hour after IL6 or IL-6/sIL-6R [13] suggesting that IL-6 might induce a state of persistent hyperexcitability which is difficult to reverse. In line with this, it has been reported that 24 hours after the injection of IL-6 into skeletal muscle an additional injection of PGE_2 _into the muscle caused stronger nociceptive behaviour than under control conditions, and it was concluded that IL-6 caused long-term *priming *of nociceptive neurones [16].

While the available data suggest that IL-6 (trans)signalling may be important for pain and inflammation, no study has investigated how neutralization of IL-6 transsignalling affects pain in an arthritis model. Therefore, in the present study we explored in the rat the effect of neutralization of IL-6 transsignalling on pain-related behaviour and inflammation in the unilateral model of antigen-induced arthritis (AIA). In order to neutralize IL-6 transsignalling, we used sgp130 which has been employed in previous studies on inflammation [2,11] and pain [13]. In the first approach, we repeatedly administered sgp130 intra-peritoneally during the course of AIA, starting six hours after arthritis induction. Employing such an approach, we previously found that the TNF-α neutralizing compounds etanercept and infliximab strongly reduced mechanical hyperalgesia at the inflamed knee joint and slightly but significantly decreased swelling at the inflamed joint [17]. In a second approach, we administered sgp130 into the knee joint together with the antigen upon arthritis induction. We analyzed pain-related and locomotor behaviour, knee swelling and, using histopathology, the severity of inflammation at the end of the observation period at 21 days after induction of arthritis.

## Materials and methods

### Antigen-induced arthritis (AIA)

Forty-five female Lewis rats (age six to eight weeks, weighing 160 to 180 g, Charles River, Sulzfeld, Germany) were used. All experiments were approved by the Thuringian state authorities and complied with EC regulations (86/609/EEC). AIA was induced as reported previously [18,19]. In brief, 500 μg methylated bovine serum albumin (m-BSA; Sigma, Deisenhofen, Germany) in saline emulsified with 500 μl Freund's complete adjuvant (Sigma; supplemented with 2 mg/ml *Mycobacterium tuberculosis *strain H37RA; Difco, Detroit, MI, USA) were injected sub-cutaneously (s.c.) twice during a one week interval for immunization. After another two weeks, m-BSA (500 μg in 50 μl saline) was injected into the left knee joint cavity to induce monoarticular AIA.

### Treatment protocols

We used recombinant human soluble gp130 (sgp130, R&D Systems, Minneapolis, MN, USA) which is effective in different species [2,4,13]. Nine animals received sgp130 intra-peritoneally (i.p.) (1 μg dissolved in 200 μl saline starting six hours after induction of AIA and on every third day until Day 12 after induction). Another nine rats were treated with sgp130 intra-articularly (i.a., 100 ng in 50 μl), which was injected simultaneously to m-BSA application. Data were compared to those from animals receiving saline i.a. (50 μl injected together with m-BSA at the time of arthritis induction), saline i.p. at the same time points as i.p.-sgp130 treatment was performed (0.9% NaCl, volume 200 μl), and to animals that were immunized, but in which no arthritis was induced (controls, each n = 9).

### Behavioural experiments

#### Pain-related behaviour

Primary hyperalgesia at the site of the inflamed knee was assessed using a dynamometer (Correx, Berne, Switzerland) as described previously [19]. In brief, increasing pressure was applied to the lateral side of the knee joint at the level of the joint space until the animals attempted to escape or vocalized. In order to quantify the antinociceptive effects of sgp130 over time, areas under the curves (AUC) depicting the changes of thresholds over time were calculated for both saline- and both sgp130-treated groups. The areas used for analyses were the integrals over the time points assessed. These were calculated using the mean of respective differences from the baseline value for each group for two consecutive time points when testing took place, for example, Days 1 and 7, multiplied with the number of days in this interval. The total area was obtained by adding the values from all intervals (1 to 3, 3 to 7, 7 to 14 and 14 to 21). The antinociceptive effect was then calculated as:

In this calculation, an antinociceptive effect of 0% means a reduction in thresholds to the same extent as in saline-treated animals, while 100% would indicate a complete return to baseline values on all testing days.

Pain-related guarding behaviour was assessed by quantification of weight bearing towards the non-inflamed hindlimb using an incapacitance tester (Linton Intrumentation, Norfolk, UK). Animals were placed in a plastic cage with both hindpaws resting on scales. The weight force on both scales was obtained and averaged for three seconds and values from three consecutive measurements were obtained for every testing day. From these values, the relative weight resting on the inflamed hindlimb was calculated as described previously [20].

Secondary hyperalgesia was assessed at sites remote from the inflamed joint, the paw and the contralateral knee joint. Mechanical secondary hyperalgesia at the contralateral knee joint was assessed as described above. In addition, secondary mechanical hyperalgesia was obtained from the paw using a dynamic plantar aesthesiometer (Ugo Basile, Comerio, Italy) as previously described [21]. This device reflects an automated form of von Frey hair testing with a blunt filament touching the paw on the plantar surface while the animal rests on a mesh floor. Then, pressure is increased until the animal withdraws its limb, and the weight force needed to elicit this response can be read out in grams. In this study, 50 g were defined as cut-off and a ramp speed of 2.5 g/s was chosen according to the procedure previously reported [22]. After allowing the animals to habituate to the device for 30 minutes, measurements were taken in triplicate over a period of approximately half an hour and means were taken as secondary mechanical hyperalgesic thresholds. Thermal secondary hyperalgesia at the hindpaws was assessed with an algesimeter (Ugo Basile, Comerio, Italy) as described previously [23,24].

#### Gait analysis

Paw prints were obtained as described previously (see [19,25]). From these prints, the distance between a print from the left (inflamed) paw and a consecutive print from the right (non-inflamed) paw (left-right-distance), mainly indicating pain [19], and the angle between consecutive paw prints, which has been associated with joint destruction [19], were assessed. For each animal and testing day, at least five gait cycles were analysed. In addition, a guarding score was assessed: 0: no guarding, 1: guarding of the hindlimb after a defined brief noxious compression of the knee, 2: visible limping during walking without previous pain stimulus, 3: no use of the hindlimb with the arthritic knee.

### Joint swelling

Swelling was assessed by measuring the mediolateral diameter of each knee using a vernier caliper (Mitutoyo, Neuss, Germany). For each animal and testing day, swelling was calculated by subtracting the diameter of the non-inflamed from the inflamed knee. In analogy to the antinociceptive effect described above, an anti-inflammatory effect was calculated, again taking into account the time course of swelling in the respective saline-treated animals. Here, areas under the swelling curves were used:

### Histopathological grading of joint inflammation and destruction

Histology of the knee joints was assessed on Day 21 after AIA induction as described previously [17,19]. Under deep anesthesia with sodium thiopentone rats were perfused with PBS and 4.0% phosphate-buffered formalin. Knee joints were removed, skinned, post-fixed in formalin, decalcified in 7% AlCl_3_, embedded in paraffin, cut into 5 μm thick frontal sections and stained with hematoxylin-eosin. Two independent observers (MG, RB) unaware of the treatment scored the sections for cellular infiltration and hyperplasia (0: no, 1: mild, 2: moderate, 3: severe alterations), cartilage destruction and bone erosion (0: no erosion, 1: erosion of < 10%, 2: of 10 to 25%, 3: of 25 to 50%, and 4: of > 50% of cartilage and bone).

### Statistical Analyses

For statistical analyses, SPSS for Windows was used (version 17.0). Data were tested for normal distribution applying Kolmogorov-Smirnov-test. Behavioural data were compared between groups using repeated measures ANOVAs with the between-subjects factor *treatment *(sgp130 i.a., sgp130 i.p., saline i.a., saline i.p.) and the within-subjects factor *time *(baseline, Days 1, 3, 7, 14 and 21 after induction of arthritis for all parameters except those from paw print analyses, that is, left-right distances and angles between paws, for which baseline and Days 7, 14 and 21 were included). Differences between treatment groups (sgp130 i.a. versus sgp130 i.p.; sgp130 i.a. versus saline i.a.; sgp130 i.p. versus saline i.p.) were analyzed for each testing day applying post-hoc t-tests.

For comparison of histological scores between groups, one-way ANOVAs were employed, followed by post-hoc *t*-tests. Antinociceptive and anti-inflammatory effects were compared between i.a.- and i.p.-sgp130-treated animals using unpaired two-sided t-tests. Significance was assumed for *P *< 0.05.

## Results

In the text, results from statistical analyses are displayed as values from multivariate testing, while figures and tables show results from post-hoc *t*-tests.

### Behavioural assessment

#### Pain-related behaviour

**Figure 1:**
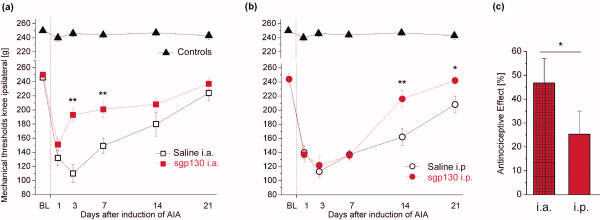
**Pain-related behaviour in the different treatment groups**. **(a) **Thresholds indicating primary mechanical hyperalgesia are significantly reduced in saline i.a.-treated animals in the acute phase of arthritis, i.a.-sgp130-treated animals show a significantly faster recovery with increased thresholds on Days 3 and 7 after induction of arthritis. **(b) **i.p.-sgp130-treated animals show a faster increase of thresholds as compared to i.p.-saline-treated animals in the chronic phase of AIA (Days 14 and 21). **(c) **Overall antinociceptive effects as calculated from areas under the curve. Data are presented as mean ± SEM. *P < 0.05; **P < 0.01; n = 9 for all groups. *Controls *in (A) and (B) show values from immunized rats without AIA induction.

Primary mechanical hyperalgesia as assessed by mechanical threshold testing at the inflamed knee joint showed a significant *time × treatment *interaction (F(15,78) = 3.743; *P *< 0.001). In particular, while saline-treated animals showed severe hyperalgesia indicated by a large reduction of mechanical pain thresholds, nociceptive thresholds were significantly increased in the i.a.-treated group on Days 3 and 7 (Figure 1a), but not in the i.p.-treated group, in which an increase in thresholds was obvious, however, in the chronic phase of AIA, that is, on Days 14 and 21 (Figure 1b). Differences between i.a.- and i.p.-sgp130-treated animals were significant on Days 3 and 7 (*P *< 0.001). The calculation of areas under the curve revealed a significantly greater overall antinociceptive effect in the i.a.-treated animals as compared to the i.p.-treated animals (*P *= 0.014; Figure 1c).

Weight bearing as a functional measure for pain-related behaviour showed a significant *time × treatment *interaction (F(15,78) = 1.900; *P *= 0.036). Here, i.a. treatment attenuated the decrease in weight resting on the inflamed hindpaw in the acute phase and accelerated the normalization of this parameter (Table 1), which was also superior to systemic treatment.

**Table 1:** Measures of secondary hyperalgesia and weight bearing

Treatment	Baseline	Day 1	Day 3	Day 7	Day 14	Day 21
*Weight bearing [% on inflamed hindlimb]*						
Saline i.p.	49.7 ± 0.7	23.5 ± 3.1	27.0 ± 1.1	31.6 ± 2.4	40.0 ± 2.6	50.3 ± 2.1
Sgp130 i.p.	49.8 ± 0.6	27.0 ± 2.6	25.3 ± 2.5	26.1 ± 3.3	40.8 ± 2.5	46.4 ± 1.7
Saline i.a.	51.1 ± 0.6	30.6 ± 3.8	30.3 ± 1.5	33.6 ± 2.8	39.2 ± 2.4	45.5 ± 1.0
Sgp130 i.a.	50.6 ± 0.8	32.4 ± 0.8 *	38.0 ± 1.7^+,^**	43.2 ± 1.3 ^+,^**	44.2 ± 1.2	48.4 ± 2.5

*Mechanical thresholds paw [g]*						
Saline i.p.	26.2 ± 2.9	16.6 ± 1.7	12.5 ± 1.5	12.2 ± 1.3	12.3 ± 1.0	14.6 ± 1.9
Sgp130 i.p.	24.6 ± 3.0	14.2 ± 2.4	16.7 ± 1.9	14.0 ± 1.1	12.3 ± 1.8	13.3 ± 1.7
Saline i.a.	22.9 ± 2.1	14.5 ± 3.7	12.8 ± 1.8	9.8 ± 2.6	9.0 ± 2.4	12.3 ± 2.4
sgp130 i.a.	24.4 ± 1.6	15.5 ± 2.0	14.5 ± 2.2	12.7 ± 1.8	15.1 ± 2.6	14.7 ± 2.1

*Thermal withdrawal thresholds paw [s]*						
Saline i.p.	15.3 ± 0.8	10.3 ± 0.9	10.3 ± 0.9	8.7 ± 0.8	8.4 ± 0.9	9.3 ± 0.9
sgp130 i.p.	14.9 ± 0.7	10.2 ± 0.8	11.3 ± 1.4	8.2 ± 1.3	9.4 ± 1.0	10.2 ± 0.9
Saline i.a.	14.9 ± 0.6	9.8 ± 0.8	9.5 ± 0.8	9.3 ± 1.1	10.1 ± 0.9	10.3 ± 1.3
sgp130 i.a.	13.4 ± 0.7	9.4 ± 0.7	10.7 ± 0.8	10.0 ± 0.7	10.5 ± 1.2	12.4 ± 1.0

Sgp, soluble glycoprotein 130; i.a., intra-articular; i.p., intra-peritoneal; data are presented as mean ± SEM. * differences between sgp130 i.p. and and sgp130 i.a.; + differences between sgp130 i.a. and saline i.a.; one symbol *P *< 0.05; two symbols *P *< 0.01.

By contrast, measures of secondary hyperalgesia assessed at the paw revealed no significant *time × treatment *interaction for mechanical thresholds (F(15,78) = 1.333; *P *= 0.218, see Table 1) or for thermal withdrawal thresholds (F(15,78) = 1.328; *P *= 0.206, see Table 1).

#### Locomotor behaviour

**Figure 2:**
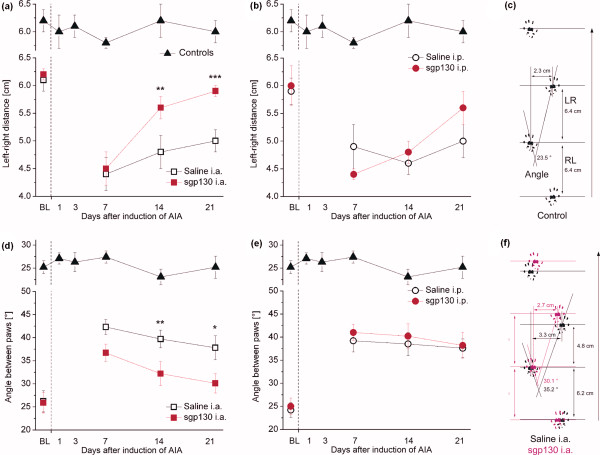
**Locomotor behaviour in the different treatment groups as assessed from paw print analysis**. **(a) **Left-right (LR) distances in i.a.-treated animals (for explanation of the parameter, see (c)), showing an attenuation of pain-related gait changes. **(b) **Left-right distances in i.p.-treated animals, showing no differences between groups. **(d), (e) **Angles between paws (for explanation, see (c)) in i.a.- (d) and i.p.- (e) treated animals, again showing a beneficial effect of i.a. sgp130 treatment. **(c), (f) **Representative specimens of paw prints from a non-inflamed (c) and a saline- or sgp130 i.a.-treated animal, respectively (f). Arrows at the right side indicate the direction of walking, LR Left-right distance, RL Right-left distance. Data in A, B, D and E are presented as mean ± SEM. *P < 0.05; **P < 0.01; ***P < 0.001; n = 9 for all groups. *Controls *in (a), (b), (d), and (e) show values from immunized rats without AIA induction.

Assessment of gait revealed no gross difference in guarding behaviour as assessed using the limping score (F(15,78) = 1.274; *P *= 0.239). However, objective gait analysis as displayed in Figure 2c (no inflammation) and 2f (i.a.-saline- and i.a.-sgp130-treated AIA animals) showed a significant *time × treatment *interaction for left-right-distance (F(9,71) = 3.812; *P *< 0.001), which has been suggested to also mainly indicate pain [19]. Here, distances were normalizing from Day 14 in the i.a.-sgp130-treated group, but not in the saline- and i.p.-treated groups (Figure 2a, b). A significant difference between sgp130-i.a. and sgp130-i.p. treatment was obvious on Day 14 only (*P *= 0.026).

Furthermore, angles between paws indicating joint destruction [19] were significantly different between groups (F(9,71) = 2.047; *P *= 0.046). Again, i.a. treatment with sgp130 attenuated the inflammation-related gait changes (Figure 2d, f), while i.p.-sgp130 application was not different from i.p.-saline treatment (Figure 2e). On Days 14 and 21, significant differences could further be obtained between sgp130-i.a.- and sgp130-i.p.-treatment (*P *= 0.012 and *P *= 0.022, respectively).

For all objective gait parameters, only Days 7 to 21 were analyzed, since only few AIA animals utilized their inflamed hindlimb in the acute phase on days 1 and 3 (saline i.a. n = 3; saline i.p. n = 3; sgp130 i.a. n = 6; sgp130 i.p. n = 4).

### Measurement of inflammation

**Figure 3:**
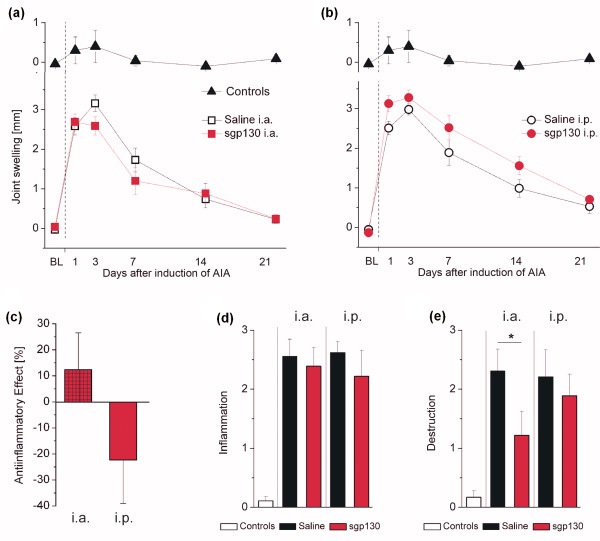
**Inflammatory changes in the different treatment groups**. Joint swelling in i.a.- **(a) **and i.p.- **(b) **treated AIA animals as compared to non-inflamed *controls*. **(c) **Anti-inflammatory effects as calculated from areas under the curve. Histopathological scores for inflammation **(d) **and cartilage and bone destruction **(e) **at day 21 of AIA, showing a beneficial effect for i.a.-sgp130-treated animals in the latter. Data are presented as mean ± SEM. *P < 0.05; n = 9 for all groups.

Joint swelling differed significantly in regard to treatment (F(15,78) = 2.166; *P *= 0.015) with lowest values being apparent in the i.a.-sgp130-treated group (Figure 3a), and rather a slight aggravation in the i.p.-treated animals (Figure 3b). Differences between i.p.- and i.a.-sgp130 treatment were significant on Days 3, 7, and 21 (*P *= 0.030, 0.011, and 0.011, respectively). Overall, the effects of either i.a.- and i.p-sgp130 treatment on joint swelling were not very pronounced, even in the i.a.-treated group, resulting in small differences in the anti-inflammatory effects as obtained from area under the curve analyses, which did not show statistical significance (*P *= 0.168; Figure 3c). Histopathological scores for inflammation at day 21 of AIA did not significantly differ between treatment groups (F = 0.174; *P *= 0.913; Figure 3d). However, scores for cartilage and bone destruction showed an effect, with least destruction in the i.a.-sgp130-treated group (F = 3.462; *P *= 0.028; Figure 3e).

## Discussion

In this study, we show that a single injection of sgp130 into the knee joint at the time of arthritis induction caused a significant long-term antinociceptive effect, although acute arthritis *per se *was barely attenuated. Antinociception is expressed as an increase of mechanical thresholds at the knee joint (reduction of hyperalgesia) and a faster normalization of pain-related gait disturbances. By contrast, repeated i.p. injection of sgp130 in the course of AIA reduced mechanical hyperalgesia only weakly, at a time point where AIA is already in the process of remission. Swelling was only weakly reduced by sgp130, but the effect of i.a. sgp130 was significantly greater than the effect of i.p. sgp130 on Day 7 of AIA. Histopathological scoring of inflammation did not show an effect of sgp130 upon either i.a. or i.p application but i.a. sgp130 produced a significant reduction of the score of cartilage and bone destruction.

The previous electrophysiological recordings from joint afferents revealed that injection of IL-6, and in particular injection of IL-6 together with its soluble receptor into the normal joint induces long-lasting sensitization for mechanical stimuli [13]. The significant antinociceptive effect of the intra-articular injection of sgp130 in the present study suggests that endogenous IL-6/sIL-6R indeed plays a significant role in the generation of arthritic joint pain. As a caveat it should be noted that sgp130 is not only restricted to sIL-6R signalling, as sgp130 also regulates the IL-6-related cytokines leukemia inhibiting factor (LIF) and oncostatin M (OSM) [5]. However, sgp130 has a lower affinity for LIF and OSM than for the IL-6/sIL-6R complex [5]. While in mice a prominent role of IL-6 in the AIA model has been established [2,26,27], the putative role of LIF and OSM in AIA is unknown. Furthermore, sgp130 is thought to prevent mainly transsignalling by IL-6/sIL-6R complexes and not to inhibit the classical IL-6 pathway [5]. Therefore, the magnitude of effects of IL-6 neutralization may be underestimated in the present study.

Both the long-lasting antinociceptive effect upon a single intra-articular injection at the time of arthritis induction and the very weak and only late effect of systemic sgp130 are remarkable. The greater effectiveness of i.a. sgp130 indicates that IL-6/sIL-6R in the joint is more important than circulating IL-6/sIL-6R. Although the dose ratio between i.a.- and i.p.-applied sgp130 was performed according to the same criteria as for etanercept in previous studies, where systemic application showed a beneficial effect [17], we cannot completely exclude that the i.p. sgp130 was underdosed and produced for this reason a less pronounced, yet detectable effect. It was pointed out that sgp130 may be present as an endogenous antagonist in the circulation and that "a molar excess of sgp130" leads to competitive inhibition of the IL-6/sIL-6R response [5]. However, not only the site of intervention (i.a. versus i.p.) may be crucial but also the timing of injection of sgp130. While the intra-articular injection of sgp130 was performed simultaneously with the injection of the antigen into the knee joint and can therefore be considered as pre-treatment, systemic sgp130 was administered for the first time six hours after induction of inflammation, that is, as post-treatment (same treatment regime as for etanercept and infliximab). These findings should be seen in the context of the effect of sgp130 on the IL-6-induced hyperexcitability. The intra-articular injection of sgp130 prevented the IL-6- or the IL-6/sIL-6R-induced sensitization upon pre-treatment but sgp130 did not reverse the IL-6- or IL-6/sIL-6R-induced hyperexcitability when it was applied after the establishment of hyperexcitability. These data suggest, therefore, that IL-6 generates a type of hyperexcitability, which is long-lasting and difficult to reverse (see Introduction).

We observed some reduction of swelling after i.a. sgp130 which may correspond to effects in previous studies in mice which showed a pronounced effect for inhibition of IL-6-transsignalling by sgp130 or splice variants thereof when applied once intra-articularly together with the antigen at the time of AIA induction [2,11]. However, histopathological scoring did not reveal a significant reduction of the inflammatory process by Day 21. By contrast, the destruction was significantly reduced which is in line with a recent study in a murine model of human TNF-mediated inflammation in which the blockade of IL-6 receptors impaired osteoclast formation and reduced bone loss, while the inflammatory process *per se *was not influenced by IL-6R-blockade [3]. This effect might even be pronounced in repeated inflammatory states. In this respect, in mice it was found that AIA can be rekindled by further injections of the antigen into the joint, and with each flare-up reaction joint destruction becomes more severe (unpublished observations).

The present and previous data show differences between the treatment with sgp130 and TNF-α-neutralization by etanercept and infliximab. First, systemic etanercept and infliximab clearly reduced mechanical hyperalgesia as well as secondary hyperalgesia at the paws at the early and late stage of AIA whereas [17,19] systemic sgp130 had only a late and weak effect. Second, injection of etanercept into the inflamed knee joint significantly reduced responses of nociceptive fibres within one hour [17], and etanercept also reduces sensitization of joint afferents by intra-articular TNF-α injection (unpublished observations) whereas intra-articular injection of sgp130 reduced the IL-6/sIL-6R-induced mechanical sensitization only in a pre-treatment approach [13]. Thus, we believe that the effects of TNF-α might overall be more reversible than those of IL-6. This might be due to its manifold putative sites of action when interfering with the pain system, that is, locally at the nerve endings [17], at the dorsal root ganglia [28], or on the spinal level [29].

## Conclusions

In addition to its pathogenetic role in chronic inflammation and bone destruction, IL-6 in the joint plays a significant role in the generation and maintenance of arthritic joint pain at acute and chronic stages of arthritis. The particular effectiveness of the intra-articular injection of sgp130 indicates that IL-6/sIL-6R (and possibly other cytokines which use the transmembrane signal-transducing subunit gp130) in the inflamed joint, rather than circulating IL-6/sIL-6R, is responsible for the generation of hyperalgesia. Furthermore, early neutralization of IL-6/sIL-6R is particularly successful in producing antinociception. The induction of pain by IL-6 or IL-6/sIL-6R is likely to result directly from an action at peripheral neurones because most peripheral nerve fibres were shown to express the transmembrane signal-transducing subunit gp130 [14,15]. Similar conclusions on the importance of the neuronal target were drawn in a study on the role of IL-6 on the generation of pathophysiological heat hyperexcitability [30]. Concerning the success of systemic treatment, we would expect that neutralization of IL-6/sIL-6R is less antinociceptive than the neutralization of TNF-α because systemic sgp130 reduced mechanical hyperalgesia much less than systemic etanercept or infliximab [17]. Finally, early neutralization of IL-6/sIL-6R by sgp130 directly at the site of joint inflammation was much more effective in the prevention of joint destruction than systemic sgp130. It may be useful, therefore, to explore clinically the effect of intra-articular injection of IL-6/sIL-6R-neutralizing compounds.

## Abbreviations

AIA: antigen-induced arthritis; ANOVA: analysis of variation; AUC: area under the curve; gp130: glycoprotein 130; i.a.: intra-articular; i.p.: intra-peritoneally; IL-6: interleukin-6; IL-6R: interleukin-6 receptor; LIF: leukemia inhibiting factor; m-BSA: methylated bovine serum albumin; OSM: oncostatin M; s.c.: sub-cutaneously; sgp130: soluble glycoprotein 130; sIL-6R: soluble form of IL-6R; TNF-α: tumour necrosis factor-α.

## Competing interests

The authors declare that they have no competing interests.

## Authors' contributions

MKB designed the study, acquired data, performed the statistical analysis, interpreted the data and wrote the manuscript. JL and DK were involved in data acquisition and statistical analysis. MG was responsible for histopathological assessment of knee joints. RB was involved in histopathological scoring and contributed expertise on the antigen-induced arthritis model. HGS designed the study, interpreted the data and wrote the manuscript.
